# The College of Nursing (Limited by Guarantee)

**Published:** 1921-03-12

**Authors:** 


### COLLEGE OF NURSING

**(Limited by Guarantee.)**

### What the Local Centres are Doing.

### LIVERPOOL CENTRE.

(Hon. Secretary, Miss Aspinall, Stanley Hospital.)

Wednesday, March 16, 7 p.m.—Lecture by Dr. E. B. Eastwood in the Lecture Hall of the Royal Infirmary. College members free; non-members, 6d.

### LONDON CENTRE.

(Secretary, Miss Bompas, 7 Henrietta Street, W. 1.)

Further Meeting to Discuss Nominations.

An important general meeting of members of the London Centre will be held on Tuesday, March 15, at the College of Ambulance, 56 Queen Anne Street, W. 1, at 8 p.m., to discuss further the procedure to be adopted in connection with the candidates nominated by members of the London Centre for the forthcoming College Council election. A vote will be taken at the meeting, and the decision arrived at will be final. All members are urged to be present in view of the importance of the question to be discussed.

Mrs. Oliver Strachey, Editor of the *Women's Leader*, will address the meeting on "Election Organisations."

The Centre "At Home."

The London Centre will be "At Home" at the Club Rooms, 7 Henrietta Street, W. 1, on Thursday, March 17, at 8 p.m. (Music and Bridge). It is hoped all new Centre members will regard this as a personal invitation, and come if possible.

### NORFOLK AND NORWICH CENTRE.

(Hon. Secretary, Miss V. A. Rutter, Albemarle House, Newmarket Road, Norwich.)

Thursday, March 17, 8 p.m.—A lecture will be given at the Eye Infirmary (by kind permission) by G. Maxted, Esq., F.R.C.S., on "The Eye and its Diseases in Relation to the Nursing Profession." Members free; non-members by tickets, price 1s. The Executive Committee will meet as usual before the lecture at 7.15 p.m. at the Eye Infirmary.

### NORTHUMBERLAND AND DURHAM CENTRE.

(Secretary, Miss M. Toyne, 17 Windsor Terrace, Jesmond.)

Friday, March 18, 6.30 p.m.—Meeting of Centre members at the Nurses' Club. As this meeting will be a very important one it is hoped members will make every effort to be present.

### SHEFFIELD CENTRE.

(Hon. Secretary, Mrs. Barnes, 34 Wilkinson Street.)

A Nomination for the Council.

At a special meeting of the members of this Centre, Miss Earle, R.R.C., Matron, Royal Hospital, Sheffield, was adopted as candidate for the forthcoming Council election. All members are asked to support Miss Earle's candidature.

Thursday, April 7.—Dr. Rupert Hallam (subject to be announced later).

Thursday, April 21.—Dr. C. W. Smith on "Cairo and its Environments," with lantern slides.

The first lecture of the above series was held on February 24, when Mr. W. S. Keer gave a most interesting and instructive address on "Points on Nursing Ear, Nose, and Throat Cases."

### YORKSHIRE CENTRE, LEEDS.

(Hon. Secretary, Miss M. V. Lindall, Hospital for Women and Children.)

Leeds Nominates its Candidate.

At a meeting of members held on Friday, March 4, at the Outlook Club, Leeds, Miss Innes, R.R.C., Lady Superintendent of the General Infirmary, Leeds, was nominated as candidate for the forthcoming Council election. Miss Innes stands for the advancement of higher education for the nursing profession. The Yorkshire Centre advises that members wishing for representation on the College Council should give their vote to this candidate to ensure her election.

Annual Meeting.

The annual meeting of the Centre has been arranged for Thursday, April 21, and so that the Hon. Secretary can prepare the ballot-papers for the meeting she requests that members will please send her nominations for officers and nine members of Committee before April 1. Names received later will not be accepted. Consent from the nominees must be given before nominations are sent. The retiring officers are eligible for re-election, with the exception of the Hon. Secretary, who has resigned, and does not seek re-election. Representatives of the following are asked: Poor-law (Leeds), health visitors (Leeds), private nursing (Leeds), Bradford, Huddersfield, Wakefield; also in place of three other members who have retired.

Miss Pressland's Death.

The Centre has sustained a great loss by the death of Miss Pressland, Matron, Clayton Hospital, Wakefield, local representative for the Centre at Wakefield. Miss Pressland was also Hon. Secretary of "The Yorkshire Group of the Association of Hospital Matrons." Both Associations will miss a loyal, helpful, and devoted fellow-worker. The members offer their appreciation and sincere and deep sympathy to Miss Pressland's relatives, and to the staff of the Clayton Hospital, Wakefield.

### EDINBURGH CENTRE.

(Hon. Secretary, Miss Cathcart, The Elms, Whitehouse Loan.)

Wednesday, March 16, 3.30 p.m.—Lecture on "The Nursing of Abdominal Cases" in the Nurses' Club, 8 Drumsheugh Gardens, by Alexander Miles, M.D., F.R.C.S.E. This lecture is open to Club as well as to Centre members.

Amusements for Club Members.

A few Club members have formed a small Amusements Committee, and they hope to arrange for gatherings to be held on the last Friday of each month. No invitations will be issued further than a notice in the nursing papers, but all members will be welcome.

A very happy evening was spent by the members of the Centre on Monday, February 28, when, after a short business meeting, followed by refreshments in the restaurant of the Club, the drawing-room floor was cleared and an hour's informal dancing was thoroughly enjoyed.

### GLASGOW CENTRE.

(Hon. Secretary, Miss E. Moseley, Western District Hospital, Oakbank.)

A meeting was held on February 26, in the Glasgow Nurses' Club (Miss Roy Reid), 10 Claremont Terrace, W., under the presidency of Miss Gregory Smith. A lecture on "Surgical Tuberculosis" was given by J. Taylor, Esq., F.R.C.S. (Edin.), Surgeon to Glasgow Corporation Hospitals for Tuberculosis. The lecturer referred to the changes which had taken place in the treatment of the disease, and detailed the most modern methods of treatment. His lecture was greatly appreciated by the large audience, and after the meeting several new members joined the Centre.

